# Incidence and risk of hypomagnesemia in advanced cancer patients treated with cetuximab: A meta-analysis

**DOI:** 10.3892/ol.2013.1301

**Published:** 2013-04-04

**Authors:** PENG CHEN, LONG WANG, HAO LI, BING LIU, ZUI ZOU

**Affiliations:** 1 Department of Stomatology, Chinese People’s Liberation Army General Hospital, Haidian, Beijing 100853;; 2 Department of Anesthesiology, The Eastern Hepatobiliary Hospital, Shanghai 200438;; 3 Department of Anesthesiology, Chinese People’s Liberation Army General Hospital, Haidian, Beijing 100853;; 4 Department of Stomatology, The General Hospital of the Air Force of the Chinese People’s Liberation Army, Beijing 100142;; 5 Department of Anesthesiology, Changzheng Hospital, Second Military Medical University, Shanghai 200003, P.R. China

**Keywords:** cetuximab, hypomagnesemia, advanced cancer, meta-analysis

## Abstract

Hypomagnesemia is a serious adverse event for patients treated with cetuximab, an inhibitor of endothelial growth factor receptor (EGFR). However, no significant association has yet been established between cetuximab and hypomagnesemia in randomized controlled clinical trials (RCTs). The present study conducted a systematic review and meta-analysis of published RCTs to assess the overall risk of hypomagnesemia associated with cetuximab. PubMed, the Cochrane Central Register of Controlled Trials, Embase and the American Society of Clinical Oncology conferences were searched for relevant RCTs. Quantitative analysis was carried out to evaluate the association between hypomagnesemia and cetuximab. A total of 7,045 patients with a variety of advanced cancers from 10 trials were included in the analysis. The overall incidence of grade 3/4 hypomagnesemia in patients receiving cetuximab was 3.9% [95% confidence interval (CI), 2.6–4.3%]. Patients treated with cetuximab had a significantly increased risk of grade 3/4 hypomagnesemia compared with patients treated with control medication, with a relative risk (RR) of 8.60 (95% CI, 5.08–14.54). Risk was observed to vary with tumor type. The study concluded that cetuximab is associated with a significant risk of hypomagnesemia in patients with advanced cancer receiving concurrent chemotherapy.

## Introduction

The endothelial growth factor receptor (EGFR), which is present in numerous cell types, is a transmembrane protein consisting of an extracellular binding domain, a hydrophobic transmembrane segment and a cytoplasmic tyrosine kinase domain, and is considered one of the best characterized ligand-receptor systems (1). Overexpression of EGFR has been identified in a variety of solid tumors (2), and EGF has played a crucial role in disease progression, poor prognosis and reduced sensitivity to chemotherapy (3). Therefore, blocking the signaling of EGF has been a major focus of new cancer therapies.

Cetuximab is a human-murine monoclonal antibody directed against the EGFR protein, which is expressed on the surface of human tumor cells (4). Cetuximab was approved by the Food and Drug Administration (FDA) for use against metastatic colorectal cancer in February 2004 (5) and first gained approval in Europe for use in the treatment of EGFR-expressing metastatic colorectal cancer following the failure of irinotecan-containing regimens (6). More recently, a meta-analysis demonstrated an improved overall survival (OS) in non-small cell lung cancer patients receiving chemotherapy plus cetuximab compared with chemotherapy alone (7). The clinical efficacy of cetuximab in a number of other malignancies, including head and neck cancer and pancreatic cancer, is currently undergoing extensive evaluation.

With the use of cetuximab, substantial adverse events have been observed. Rashes, diarrhea, fatigue, neutropenia, hypertension, nausea, infusion-related or hypersensitivity reactions, and hand-foot skin reactions were extremely common when cetuximab was first administrated for advanced cancer. In September 2005, the FDA released a warning about the possibility of severe hypomagnesemia in relation to cetuximab therapy (8). A large number of patients with metastatic colorectal cancer receiving cetuximab developed severe hypomagnesemia that was refractory to oral magnesium supplementation (9,10). However, no significant association has yet been established between cetuximab and hypomagnesemia in randomized controlled clinical trials (RCTs). Thus, we undertook a systematic review of the relevant RCTs to evaluate the risk of hypomagnesemia associated with cetuximab treatment for advanced cancer.

## Materials and methods

### Data source

An extensive search of PubMed (up to March, 2012), the Cochrane Central Register of Controlled Trials (up to Cochrane Library Issue 3, 2012), and Embase (up to March, 2012) was conducted to identify relevant RCTs for the meta-analysis, using the keywords; ‘cetuximab’, ‘erbitux’, ‘cancer’ and ‘hypomagnesemia’. Abstracts and virtual meeting presentations from the American Society of Clinical Oncology conferences held between January 2000 and March 2012 were also searched for relevant RCTs. The reference lists of articles, reviews, letters to the editor and case reports were also searched to find those not yet included in the computerized databases. The language of the research papers was not restricted.

### Study selection

RCTs that directly compared advanced cancer patients treated with and without cetuximab, respectively, were selected for the analysis. Phase I and single-arm phase II trials were excluded due to the lack of control groups. Specifically, clinical trials that met the following criteria were included in the meta-analysis: i) prospective phase II and phase III RCTs in patients with advanced cancer; ii) random assignment of participants to cetuximab treatment or control group (placebo or best supportive care), in addition to concurrent chemotherapy and/or treatment with a biological agent; and iii) available data, including events or incidences of hypomagnesemia and sample size for analysis.

### Data extraction

Two researchers independently extracted data from each identified trial using a predesigned review form. The following data were included: authors of each study, publication year, trial design, number of patients, number of patients eligible for hypomagnesemia evaluation, age, gender, intervention, dose of cetuximab administered, cancer type, phase of trial, follow-up time, allocation concealment, blinded analysis and events or incidences of hypomagnesemia.

### Qualitative assessment

The studies were appraised independently by two authors based on the standard criteria (randomization, blinding, loss to follow-up and generation of allocation concealment), and additional quantitative quality was assessed using the scoring system developed by Jadad *et al*(11), appropriately modified according to the treatments under study. The quality scoring system was as follows: i) adequacy of randomization, coded as properly used with detailed description of randomization (score 2), randomized but details not reported (score 1) and inappropriate randomization (score 0); ii) allocation concealment, coded as properly used (score 2), unclear (score 1) and not used (score 0); iii) blinded method, coded as double blind (score 2), single-blind (score 1) and open label or unclear (score 0); iv) drop-outs and follow-ups, coded as data given (score 1), and data not given (score 0). Any disagreement was resolved by discussion.

### Clinical end-points

The primary end-point was the incidence of hypomagnesemia. Hypomagnesemia in these studies was assessed and recorded according to the Common Terminology Criteria for Adverse Events (version 2 or 3) (12,13).

### Statistical analysis

Stata version 10.0 software (StataCorp., College Station, TX, USA) was used for the statistical analysis. The incidence of hypomagnesemia was calculated using the number of patients with hypomagnesemia in the cetuximab group and the total number of patients receiving cetuximab treatment. The proportion of patients with hypomagnesemia was calculated and the 95% confidence interval (CI) was derived for each trial.

The Chi-square test of heterogeneity and the I^2^ measure of inconsistency were used to assess the heterogeneity between trials. With an I^2^ value of >50% indicating significant heterogeneity, the following techniques were used as explanations: (a) subgroup analysis; (b) sensitivity analysis performed by excluding the trials which potentially biased the results; and (c) the random effects model was used to explore the cause of the heterogeneity. The Begg’s test was used to determine the presence of publication bias with regard to the primary variable [relative risk (RR) of hypomagnesemia]. A two-tailed P-value of <0.05 was considered to indicate a statistically significant difference.

## Results

### Identification of included studies

**Figure 1:**
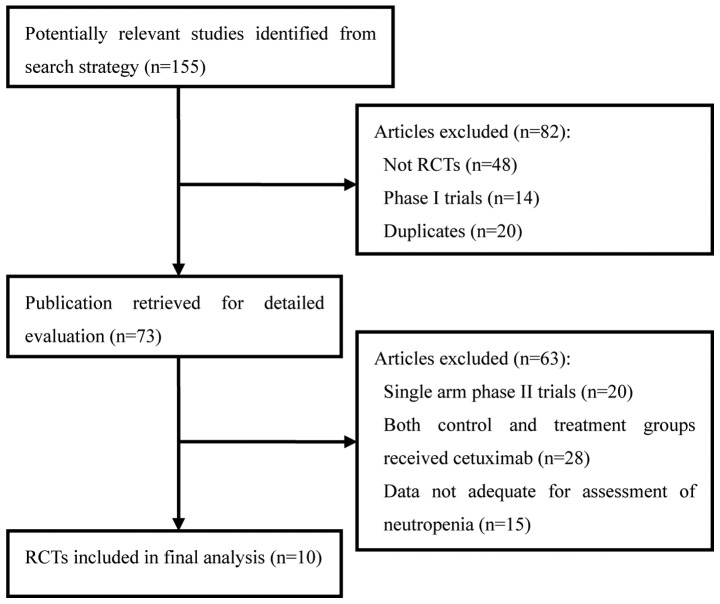
Selection process for RCTs included in the meta-analysis. RCTs, randomized controlled clinical trials.

A total of 155 clinical studies relevant to cetuximab were identified by the literature search. Review articles, case reports, meta-analyses, observational studies (n=48), phase I studies (n=14), single-arm phase II studies (n=20), duplicates (n=20), studies in which the control and treatment groups each received cetuximab (n=28) and those data not adequate for assessment of severe neutropenia (n=15; Fig. 1) were excluded. Ultimately, 10 RCTs, including five phase II and five phase III studies, were selected for analysis, involving a total of 7,045 patients. The main characteristics (type of study design, underlying malignancy of included patients, concurrent treatment and number of patients) of the 10 included RCTs are presented in Table I. Randomized treatment allocation sequences were generated in all trials. Only one trial was double-blinded and placebo-controlled (14), five of the trials were open-label (15–19) and four trials were not specified (20–23). All trials reported the number and reason of withdrawals and drop-outs. None mentioned allocation concealment. A total of seven trials were described as multicenter trials and three did not mention their status (19,20,23). The median follow-up time for four of the studies (18,19,21,22) ranged from 6.8 to 31 months, while six studies did not state this factor. Hypomagnesemia was assessed and recorded according to the National Cancer Institute’s Common Toxicity Criteria, version 2 or 3 (12,13). The baseline Eastern Cooperative Oncology Group (ECOG) performance status of all patients was between 0 and 2. Patients were required to have adequate hepatic, renal and hematological function. The underlying malignancies observed consisted of colorectal cancer (six studies), non-small cell lung cancer (two studies) and head and neck cancer (two studies).

### Risk of hypomagnesemia for cetuximab administration

**Figure 2:**
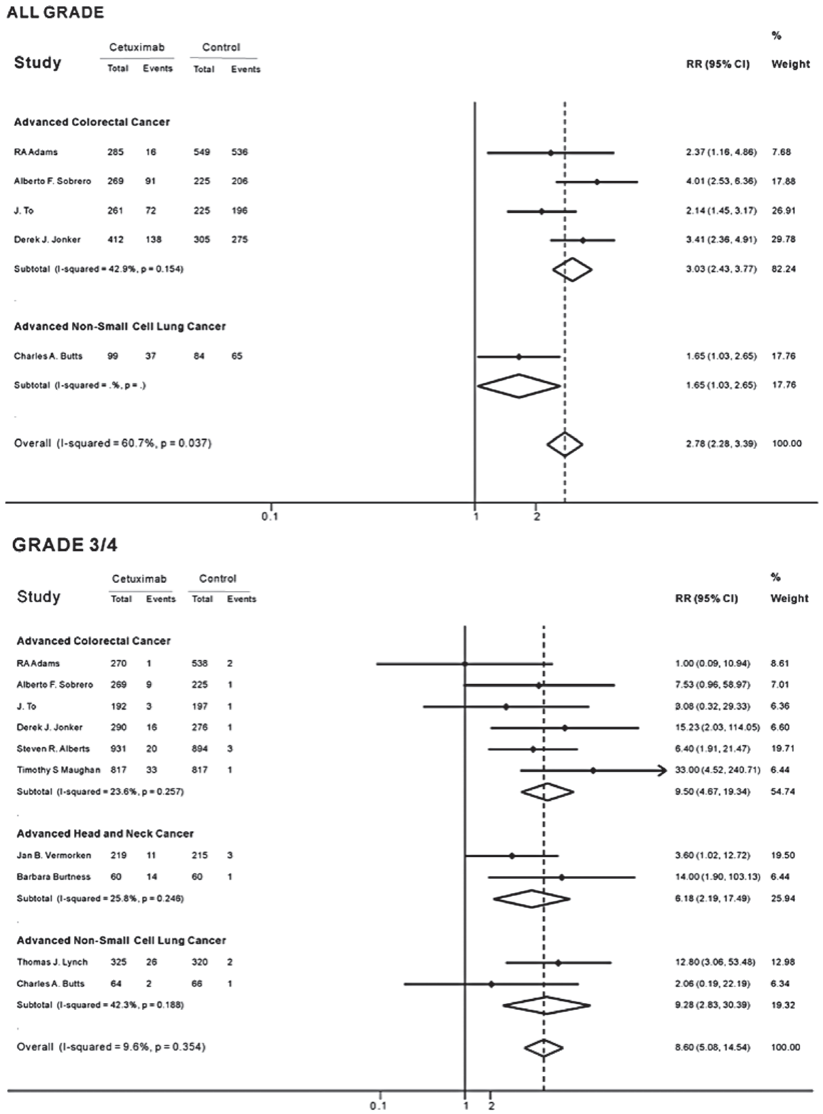
Relative risk (RR) of hypomagnesemia associated with cetuximab treatment compared with control treatment. RR<1, numerically lower incidence than control chemotherapy; RR>1, numerically higher incidence than control chemotherapy. If 95% CI does not include the number 1 it demonstrates a significant difference between the two groups (P<0.05).

As no heterogeneity was found among the included studies in the overall analysis (all-grades of hypomagnesemia I^2^, 60.7%, P=0.037; grade 3/4 I^2^, 9.6%, P=0.354), the fixed-effects model was used. The overall RR of grade 3/4 hypomagnesemia with cetuximab versus control was 8.60 (95% CI, 5.08–14.54; Fig. 2), indicating a significantly higher incidence of grade 3/4 hypomagnesemia in the cetuximab groups. The RR of the subgroup analysis suggested a significant association between grade 3/4 hypomagnesemia and cetuximab treatment among patients with non-small cell lung cancer (RR, 9.28; 95% CI, 2.83–30.39; Fig. 2). The RR of grade 3/4 hypomagnesemia was lowest in patients with head and neck cancer treated with cetuximab compared with controls (RR, 6.18; 95% CI, 2.19–17.49), and highest in patients with colorectal cancer (RR, 9.50; 95% CI, 4.67–19.34). Of all the trials, five reported that the cetuximab groups had a higher incidence of grade 3/4 hypomagnesemia compared with the control groups.

### Incidence of hypomagnesemia for cetuximab administration

The overall incidence of grade 3/4 hypomagnesemia in the patients receiving cetuximab was 3.9% (95% CI, 2.6–4.3). Patients with differing tumors may be at varying risks of grade 3/4 hypomagnesemia due to differences in tumor malignancy and the associated treatments. The present study explored whether having a specific type of cancer was associated with a higher risk of severe neutropenia compared with other cancers. As shown in Table II, the risk of grade 3/4 hypomagnesemia varied according to the tumor type. The highest incidence of grade 3/4 hypomagnesemia was observed in patients with non-small cell lung cancer (9.0%; 95% CI, 5.0–15.4), while the lowest incidence was observed in patients with colorectal cancer (2.9%; 95% CI, 1.7–3.1).

### Publication bias

No publication bias was detected for the primary variable of the present study (RR of grade 3/4 hypomagnesemia) by Begg’s and Egger’s tests (P=0.38; P=0.29, respectively).

## Discussion

Hypomagnesemia may result in cardiac arrhythmia, coronary artery vasospasm and sudden cardiac death. Adequate management of hypomagnesemia is important for the numerous patients who receive cetuximab-based therapy. However, the symptoms of hypomagnesemia may be fairly non-specific, including irritability, paresthesia and severe fatigue, which may easily be attributed to the underlying tumor or to previous chemotherapy regimens (24). Hypomagnesemia is often ignored in studies, and serum magnesium levels should be monitored better when cetuximab-based therapy is performed for advanced cancer. In RCTs discussing the association of hypomagnesemia and cetuximab, an individual RCT is not powerful enough to detect a significant correlation; therefore the contribution of cetuximab to the development of hypomagnesemia is difficult to assess. The present study combined 10 RCTs to overcome this limitation. The result demonstrated a high incidence of grade 3/4 hypomagnesemia (3.9%; 95% CI, 2.6–4.3) associated with cetuximab treatment for advanced cancer. Cetuximab treatment had a higher risk of grade 3/4 hypomagnesemia compared with the control (RR, 8.60; 95% CI, 5.08–14.54). The present study also showed that the risk of grade 3/4 hypomagnesemia with cetuximab may vary with the tumor type. Patients with advanced colorectal cancer had the highest incidence of grade 3/4 hypomagnesemia.

The mechanisms behind this toxicity have not been well defined. Numerous studies on hereditary renal Mg^2+^-wasting syndromes and inborn errors of the Mg^2+^ balance demonstrated that several new proteins were involved in transepithelial Mg^2+^ transport in the distal convoluted tubule, including the Mg^2+^-permeable channel TRPM6 (transient receptor potential cation channel, subfamily M, member 6) and TRPM7 (25–27). Groenestege *et al*(28) revealed that *in vitro* cetuximab preincubation abolished the stimulatory effect of EGF on TRPM6 activity. Moreover, EGFR is highly expressed in the kidney, particularly in the ascending limb of the loop of Henle, where 70% of filtered Mg^2+^ is reabsorbed. Cetuximab, as an EGFR blockade, may affect Mg^2+^ transport (29). However, this effect has not been described with other small molecule anti-EGFR agents, such as gefitinib and erlotinib. Thus, a pure anti-EGFR effect does not adequately explain this toxicity. Recent data from panitumumab clinical trials have also reported hypomagnesemia toxicity in patients with metastatic colorectal cancer (30). This suggests that hypomagnesemia toxicity is a monoclonal antibody anti-EGFR-specific phenomenon.

There are several limitations in the present study analysis that require consideration. Firstly, the meta-analysis results are affected by clinical heterogeneity. The trials have varying patient clinical profiles, concurrent chemotherapies, lengths of follow-up and lengths of treatment; thus, differences among trials are inevitable, and there is always some heterogeneity, even within individual trials. However, heterogeneity does not necessarily preclude pooling of the results since individual patients are only directly compared with other patients within the same trial and not across trials (31,32). Given the uncertainty resulting from this clinical heterogeneity, subgroup analyses were performed in the present meta-analysis. Secondly, the meta-analysis only included 10 studies out of 155 identified in the literature search. In this regard, only those trials conducted with a rigorous methodology were selected in order to provide solid conclusions. Meta-analyses often include small numbers of studies and heterogeneity is therefore a necessary consequence. Higgins *et al* evaluated Cochrane reviews and identified that 67% included five studies and that 20% included ten studies (33). A lower threshold for the number of studies to be included in a meta-analysis has not yet been established. Finally, not all articles had data available on all grades of hypomagnesemia.

In conclusion, the present study showed that cetuximab is associated with a significant risk of hypomagnesemia in patients with advanced cancer who were receiving concurrent chemotherapy. This risk varies with the tumor type. Early monitoring of hypomagnesemia is important when cetuximab-based therapy is performed. Patients undergoing cetuximab administration with grade 3/4 of hypomagnesemia should receive appropriate and aggressive replacement therapy due to the high risk of cardiac arrhythmias and sudden mortality.

## Figures and Tables

**Table I:** Characteristics of randomized controlled clinical trials (RCTs) included in the meta-analysis.

First author (ref.)	Trial phase	Number of patients enrolled	Number for analysis	Underlying malignancy	Concurrent treatment	Jaded score	Cetuximab dose (mg/m^2^ per week)
Tol (21)	III	389	389	Colorectal cancer	Capecitabine, oxaliplatin and bevacizumab	3	250
Lynch (15)	III	676	645	Non-small cell lung cancer	Paclitaxel or docetaxel	3	250
Jonker (19)	III	572	566	Colorectal cancer	Fluoropyrimidine, irinotecan and oxaliplatin		250
Alberts (22)	III	2686	1825	Colorectal cancer	Fluorouracil, leucovorin and irinotecan	4	250
Maughan (23)	III	1634	1634	Colorectal cancer	Oxaliplatin and fuoropyrimidine	3	250
Adams (16)	III	804	804	Colorectal cancer	Leucovorin, oxaliplatin and iluorouracil or oxaliplatin and capecitabine		250
Vermorken (20)	III	442	434	Head and neck cancer	Fluorouracil, cisplatin or carboplatin	4	250
Sobrero (17)	III	1298	1267	Colorectal cancer	Irinotecan	3	250
Butts (18)	II	131	130	Non-small cell lung cancer	Cisplatin or carboplatin	3	250
Burtness (14)	III	117	116	Head and neck cancer	Cisplatin and placebo	5	125

**Table II:** Incidence of grade 3/4 hypomagnesemia with cetuximab among patients with various tumor types.

Tumor type	Number of studies	Cetuximab	Control	Incidence (95% CI)
Overall	10	135 (3437)	16 (3608)	0.039 (0.026–0.043)
Colorectal cancer	6	82 (2769)	9 (2947)	0.029 (0.017–0.031)
Non-small cell lung cancer	2	25 (279)	4 (275)	0.090 (0.050–0.154)
Head and neck cancer	2	28 (389)	3 (386)	0.07 (0.015–0.1 77)

Data are presented as number of patients with grade 3/4 hypomagnesemia (number included in the present study). CI, confidence interval.
